# The EORTC QLQ43 and FACT H&N questionnaires of quality of life at 1 and 5 years after treatment and dental care in head and neck cancer patients: a pilot study

**DOI:** 10.1007/s12094-024-03567-5

**Published:** 2024-06-23

**Authors:** Marc Guedea, Meritxell Sánchez, Alicia Lozano, Montse Ferrer, Angels Pont, Ferran Guedea, Sandra Clotet, Marc Juárez, Pablo Araguas, Montse Ventura, Nuno Gustavo d’Oliveira, Josep Maria Ustrell

**Affiliations:** 1 Orthodontics Department, Facultat de Medicina i Ciències de la Salut, University of Barcelona (UB), Barcelona, Spain; 2 Radiation Oncology Department, Institut Català d’Oncologia (ICO), Hospital Duran I Reynals, L’Hospitalet de Llobregat, Barcelona, Spain; 3 Head and Neck Cancer Tumor Board, Institut Català d’Oncologia (ICO), Hospital Duran i Reynals, L’Hospitalet de Llobregat, Barcelona, Spain; 4 Health Services Research Group, Hospital del Mar Research Institut – IMIM, Barcelona, Spain; 5 CIBER Epidemiología y Salud Pública (CIBERESP), Universitat Pompeu Fabra, Barcelona, Spain; 6 Clinical Sciences Departament, Facultat de Medicina i Ciències de la Salut. University of Barcelona (UB), L’Hospitalet de Llobregat, Barcelona, Spain; 7 University of Barcelona (UB), Barcelona, Spain

**Keywords:** Health-related quality of life, EORTC QLQ-H and N43, FACT H and N, Dental care, Head and neck cancer, Radiotherapy

## Abstract

**Purpose:**

This study aimed to examine health-related quality of life (HRQoL) in head and neck cancer patients at 1 and 5 years after successful treatment of their tumors, and to explore the usefulness of 2 instruments for assessing the need of dental care services.

**Methods:**

This cross-sectional pilot study included 20 adult patients with head and neck cancer who completed the Functional Assessment of Cancer Therapy-Head and Neck (FACT H&N) Symptom Index and the European Organization for Research and Treatment of Cancer Quality of Life Head and Neck Module (EORTC QLQ-H&N43) after 1 and 5 years of treatment.

**Results:**

Mean (standard deviation, SD) scores of the FACT H&N Symptom Index were higher (better HRQoL) at 5 years than at 1 year (24.1 [4.4] vs. 21.1 [6.4]; *p* = 0.236). Only three of the ten items of FACT H&N (swallow, pain in mouth/throat or neck, and solid foods) evaluated oral health. In the EORTC QLQ-H&N43 questionnaire, scores were lower at 5 years (better HRQoL) in almost all multi- and single-item symptoms. This questionnaire includes four multi-item scales (pain in the mouth, social eating, swallowing, and problems with teeth) measuring dental and orthodontic needs.

**Conclusion:**

HRQoL in patients with head and neck cancer improved with the length of follow-up. The EORTC QLQ-H&N43 has more items addressing oral health compared to the FACT H&N Symptom Index and may be more adequate to assess the need of dental therapy in clinical practice.

## Introduction

Head and neck cancer includes malignant epithelial tumors arising in the oral cavity, paranasal sinuses, nasal cavity, pharynx, and larynx [1]. Globally, head and neck cancer ranks seventh among all cancers, with 325,000 deaths annually and approximately 660,000 newly diagnosed cases [2]. In Spain, head and neck cancer represents 5% of all oncological cases in adults [3]. Smoking and alcohol use are well-known risk factors for head and neck cancer, and more recently human papillomavirus infection (HPV) has been added as risk factor for cancer of the oropharyngeal region [4]. Although the incidence of head and neck cancer is slowly declined globally, partly because of decreased smoking, cases of HPV-associated oropharyngeal cancer are increasing [4].

Patients with head and neck cancer should be managed by a multidisciplinary team of specialists in different oncological areas, given that surgery, radiation therapy, and systemic chemotherapy are major pillars of treatment. The usual schedule of radiation therapy includes 60– 70 Gy, with 5 day week delivery. Cisplatin/carboplatin or cetuximab are commonly used concomitantly as radio-sensitizers. Targeted immunotherapy has been recently added to the therapeutic landscape of head and neck tumors in selected patients [5].

As a result of remarkable advances in the management of patients with head and neck cancer, odontologists are faced with an increasing number of cancer survivors seeking dental care to achieve optimal dental function and aesthetics. Although clinical assessment of oral health is an indispensable step in the diagnostic work-up studies, evaluation of health-related quality of life (HRQoL) is rarely performed. In fact, HRQoL instruments have been applied widely in oncology but very few in dental care services. The two main HRQoL instruments specifically designed for head and neck malignancies are the Functional Assessment of Cancer Therapy in Head and Neck (FACT H&N) [6] and the European Organization for Research and Treatment of Cancer Quality of Life-Head and Neck Cancer (EORTC QLQ- H&N43) [7, 8].

In recent years, HRQoL has been a dimension increasingly considered in long survivors of head and neck cancer patients as an important measure of the effect of the quality of oral health in daily living. However, the benefits of dental care after the end of cancer treatment and the impact on the HRQoL of patients in the post-treatment phase remains poorly defined. It is not clear to what extent HRQoL instruments developed in the oncology setting could be also appropriate for use in dental care services. Therefore, the aim of this pilot study was to examine HRQoL in head and neck cancer patients at 1 and 5 years after successful treatment of their tumors, as well as to explore the usefulness of the FACT H&N and EORTC QLQ-H&N43 instruments for assessing the need of dental care services.

## Materials and methods

### Design and participants

A cross-sectional pilot study was conducted in 20 head and neck cancer patients, who were evaluated in 2023 over their follow-up after 1 and 5 years of successful treatment of their malignancies. All of these patients had been diagnosed and treated at Institut Català d’Oncologia (ICO) in L’Hospitalet de Llobregat, Barcelona (Spain). ICO is a public and monographic cancer center of reference for more than 50% of the adult population of Catalonia.

Inclusion criteria were patients aged ≥ 18 years who had been diagnosed with head and neck cancer at the ICO undergoing radical radiotherapy with or without chemotherapy. These patients presented with tumors located in the oral cavity, hypopharynx, oropharynx, and nasopharynx. Patients with primary laryngeal tumors, neoplasms located in other regions of the body, and those who did not receive radiotherapy as the main cancer treatment were excluded.

The study was approved by the Clinical Research Ethics Committee (CEIC) of Institut Català d’Oncologia (ICO) and Hospital Universitari de Bellvitge (code PR354/22, approval date February 9, 2023), L’Hospitalet de Llobregat, Barcelona, Spain. All patients provided the signed written informed consent.

### Study procedures

Patients underwent a computer-assisted telephone interview administered by a trained and experienced interviewer who administered the two study questionnaires, the FACT H&N Symptom Index and the EORTC QLQ-H&N43.

The FACT H&N, a 39-item HRQoL questionnaire designed for patients with head and neck cancer, is comprised of the FACT-General, which includes the physical well-being, social/family well-being, emotional well-being, and functional well-being subscales, and the Head and Neck Cancer Subscale. The FACT Head & Neck Symptom Index derived from the FACT H&N, comprises the 10 items measuring the disease symptoms of most interest [6]. Like all FACT scales, it uses a 7 day response period and a 5-point Likert-type response scale (not at all, a little bit, somewhat, quite a bit, and very much), and the index score ranges from 0 to 100 where higher scores indicate more serious symptoms.

The EORTC QLQ-H&N43 is a revised and updated version of the EORTC QLQ H&N35 [7, 8]. The questionnaire includes 12 multi-item scales (dry mouth/sticky saliva, pain in the mouth, senses, social eating, swallowing, sexuality, body image, speech problems, problems with teeth, anxiety, shoulder problems, and skin problems) and 7 single-item symptom scales (coughing, opening mouth, social contact, neurological problems, swelling in the neck, weight loss, and problems with wound healing). All items have a Likert-like response format (not at all = 1; a little = 2; quite a bit = 3; and very much = 4). Scores for all scales and single items are calculated by linear transformation of raw scores into a 0–100 score, with 100 representing heavy sympton burden. In the international validation of the EORTC QLQ-H&N43 questionnaire, Spain was one of the 18 countries included [8].

In addition to completing the HRQoL questionnaires, other data collected were demographic variables (age, sex); smoking status; alcohol consumption; tumor location; histological grade; tumor and lymph nodal stage; total radiation dose; surgical treatment; and chemotherapy (including cycles of cisplatin).

### Study endpoints

The primary endpoint was to determine differences in HRQoL measured using the FACT H&N Symptom Index and EORTC QLQ-H&N43 questionnaires in patients with head and neck cancer at 1 and 5 years after completion of cancer treatment. Secondary endpoints were the following: a) to determine which HRQoL questionnaire would be more appropriate for evaluating potential dental and orthodontic needs of these patients, and b) to establish the feasibility of the administration of the HRQoL instruments by a telephone interview.

### Statistical analysis

Categorical variables are presented as absolute numbers and percentages, and quantitative data as mean and standard deviation (SD). Data of the FACT H&N and the EORTC QLQ-H&N43 were analyzed at dimension and item levels. At dimension level, scores of patients at 1 and 5 years after the end of cancer treatment were compared using the Student’s *t* test. At item level, the Likert scales of both HRQoL questionnaires were dichotomized into two categories: “not at all” and all other response options, and the percentages of responses at 1 and 5 years were compared with the chi-square test. A *p* value < 0.05 was established as statistically significant.

## Results

### Characteristics of patients

Of the 20 eligible patients, 10 had completed their oncological treatment for head and neck cancer since 1 year and the remaining 10 patients since 5 years. Salient clinical data of the study patients are shown in Table 1. Most patients were men, with a mean age of 61 years, and tumors of the oropharynx as the most common location. The most frequent stage was T2 and T3 among patients evaluated at 1 year and 5 years, respectively (Table 1).

**Table 1:** Clinical characteristics of the study population

Variables	Time after completing of oncological treatment		*p* value
	1 year (*n* = 10)	5 years (*n* = 10)	
Sex, *n* (%)			
Men	7 (70)	8 (80)	0.606
Women	3 (30)	2 (20)	
Age, years, mean (SD)	61.7 (9.9)	60.6 (10.9)	0.815
Smoking habit, *n* (%)			
Current smoker	3 (30)	3 (30)	0.856
Ex-smoker	2 (20)	3 (30)	
Never smoker	5 (50)	4 (40)	
Alcohol consumption, *n* (%)			
Never	6 (60)	5 (50)	0.232
Occasional	2 (20)	3 (30)	
Heavy	0	2 (20)	
Missing	2 (20)		
Primary cancer site, *n* (%)			
Hypopharynx	1 (10)	3 (30)	0.485
Nasopharynx	1 (10)	2 (20)	
Oral cavity	3 (30)	1 (10)	
Oropharynx	5 (50)	4 (40)	
Tumor grade, *n* (%)			
Grade 1	2 (20)	1 (10)	0.343
Grade 2	2 (20)	4 (40)	
Unknown	6 (60)	5 (50)	
Tumor stage, *n* (%)			
T1	2 (20)	3 (30)	0.399
T2	4 (40)	1 (10)	
T3	2 (20)	4 (40)	
T4	1 (10)	2 (20)	
Unknown	1 (10)	0	
Nodal stage, *n* (%)			
N0	3 (30)	4 (40)	0.807
N1	1 (10)	2 (20)	
N2	5 (50)	3 (30)	
N3	1 (10)	1 (10)	
Radiation dose, Gy, mean (SD)	67.2 (4.1)	68.0 (4.2)	0.672
Surgical tumor excision, *n* (%)	4 (40)	4 (40)	1.0
Chemotherapy, *n* (%)	7 (70)	7 (70)	1.0
Cisplatin	5 (50)	6 (60)	0.637
Cycles of cisplatin, mean (SD)	5.0 (2.0)	2.5 (0.6)	0.053

*SD* standard deviation

Patients were treated with radical radiotherapy with volumetric modulated arc therapy (VMAT) once a day, 5 times each week for 7 weeks, in 33 sessions, with a total dose between 60 and 70 Gy on the tumor (2–2.12 Gy per fraction) and 54.12 Gy on lymph node areas at risk of subclinical disease (1.64 Gy per fraction). Chemotherapy (cisplatin-based in half of the patients) was also administered in 70% of patients concomitant with radical radiotherapy. The distribution of clinical variables was similar among patients in the 1 and 5 years groups, without statistically significant differences.

### Quality of life assessed with the FACT H and N Symptom Index

The mean (SD) total score was higher in the group of patients evaluated at 5 years than in those evaluated at 1 year (better HRQoL at 5 years) (24.1 [4.4] vs. 21.1 [6.4]), although differences were not statistically significant (*p* = 0.236). Table 2 shows the percentage of patients who reported “a little bit”, “somewhat”, “quite a bit” and “very much” in different items of the questionnaire. Among patients evaluated after 1 year of treatment, the item I worry that my condition will get worse was present in the highest proportion of patients followed by I have pain (80%), and I am able to communicate with others (80%). In the group of patients evaluated at 5 years after oncological treatment, three items (I worry that my condition will get worse, I am able to communicate with others, and I can eat solid foods) were present in 90% of patients. Only three items of the instrument were focused on oral health: I can swallow naturally and easily, I have pain in my mouth, throat or neck, and I can eat solid foods.

**Table 2:** Total FACT H&N Symptom Index score and patients reporting problems after 1 and 5 years of treatment

Variables	Time after completing of oncological treatment		*p* value
	1 year (*n* = 10)	5 years (*n* = 10)	
Total score, mean (SD)	21.1 (6.4)	24.1 (4.4)	0.236
Symptom, *n* (%)*			
* I have pain*	8 (80)	7 (70)	0.606
* I have a lack of energy*	7 (70)	7 (70)	1.0
* I can swallow naturally and easily*	6 (60)	8 (80)	0.329
* I have pain in my mouth, throat or neck*	7 (70)	5 (50)	0.361
* I have trouble breathing*	4 (40)	5 (50)	0.653
* I am able to communicate with others*	8 (80)	90 (90)	0.531
* I have nausea*	3 (30)	2 (20)	0.606
* I can eat solid foods*	7 (70)	90 (90)	0.264
* I worry that may condition will get worse*	90 (90)	90 (90)	1.0
* I am content with the quality of my life right now*	6 (60)	3 (30)	0.178

*Number of patients reporting all response options except for “not at all”

### Quality of life assessed with the EORTC QLQ-H and N43 Questionnaire

The mean scores of the general core multi-items and single-item symptom scales in patients after 1 and 5 years of completing oncological treatment are shown in Table 3. This table also includes the number and percentage of patients reporting problems rated as “little”, “quite a bit”, and “very much” for each item of the questionnaire. Statistically significant differences with worse HRQoL at 1 year as compared to 5 years were found in the mean (SD) scores of the multi-item domains of anxiety (61.7 [38.5] vs. 30.0 [18.9], p = 0.036), shoulder problems (41.7 [41.8] vs. 8.3 [16.2], p = 0.037) and neurological problems (50.0 [42.3] vs. 13.3 [28.1], p = 0.035), as well as in the symptoms of “sticky saliva” (70% vs. 20%, p = 0.025), “problems eating in front of the family” (40% vs. 0%, p = 0.025), “tingling or numbness in hand and feet” (70% vs. 20%, p = 0.025), and “worried that the weight is too low” (40% vs. 0%, p = 0.025).

**Table 3:** Results of the EORTC QLQ-H&N43 questionnaire after 1 and 5 years of treatment

Variables	Time after completing oncological treatment		*p* value
	1 year (*n* = 10)	5 years (*n* = 10)	
Multi-item scales			
M1: Dry mouth/sticky saliva, mean (SD) score	65.0 (34.6)	43.3 (36.2)	0.188
* Have you had a dry mouth?*	9 (90)	8 (80)	0.531
* Have you had sticky saliva?*	7 (70)	2 (20)	0.025
M2: Pain in the mouth, mean (SD)	24.2 (22.0)	10.8 (12.5)	0.113
* Have you had pain in your mouth?*	6 (60)	1 (10)	0.019
* Have you had pain in your jaw?*	5 (50)	2 (20)	0.160
* Have you had soreness in your mouth?*	7 (70)	4 (40)	0.176
* Have you had pain in your throat?*	3 (30)	4 (40)	0.639
M3: Problems with senses, mean (SD) score	51.7 (39.6)	21.7 (28.4)	0.067
* Have you had problems with your sense of smell?*	4 (40)	3 (30)	0.639
* Have you had problems with your sense of taste?*	8 (80)	5 (50)	0.160
M4: Social eating, mean (SD) score	32.5 (36.5)	18.3 (22.8)	0.312
* Have you had problems eating?*	5 (50)	7 (70)	0.361
* Have you had problems eating in front of your family?*	4 (40)	0	0.025
* Have you had problems eating in front of other people?*	4 (40)	2 (20)	0.329
* Have you had problems enjoying your meals?*	5 (50)	3 (30)	0.361
M5: Swallowing, mean (SD) score	25.0 (22.9)	16.7 (17.6)	0.373
* Have you had problems swallowing liquids?*	2 (20)	2 (20)	1.0
* Have you had problems swallowing pureed food?*	3 (30)	1 (10)	0.264
* Have you had problems swallowing solid food?*	7 (70)	6 (60)	0.639
* Have you choked when swallowing?*	7 (70)	4 (40)	0.178
M6: Sexuality, mean (SD) score	51.7 (41.9)	35.0 (39.6)	0.373
* Have you felt less interest in sex?*	6 (60)	4 (40)	0.371
* Have you felt less sexual enjoyment?*	7 (70)	5 (50)	0.361
M7: Body image, mean (SD) score	41.1 (45.7)	10.0 (14.3)	0.065
* Have you had problems with your appearance?*	4 (40)	4 (40)	1.0
* Have you felt less physically attractive as a result of your disease or treatment?*	5 (50)	1 (10)	0.051
* Have you felt dissatisfied with your body?*	5 (50)	2 (20)	0.160
M8: Speech problems, mean (SD) score	14.7 (12.5)	19.3 (16.8)	0.489
* Have you had problems with hoarseness?*	5 (50)	9 (90)	0.051
* Have you had problems taking to other people?*	1 (10)	2 (20)	0.531
* Have you had problems taking on the telephone?*	1 (10)	1 (10)	1.0
* Have you had problems in a noisy environment?*	5 (50)	6 (60)	0.653
* Have you had problems speaking clearly?*	3 (30)	4 (40)	0.639
M9: Problems with teeth, mean (SD) score	44.4 (31.9)	33.3 (30.1)	0.433
* Have you had problems with your teeth?*	7 (70)	5 (50)	0.361
* Have you had problems because of losing some teeth?*	5 (50)	4 (40)	0.653
*Have you had problems chewing?*	7 (70)	6 (60)	0.639
M10: Anxiety, mean (SD) score	61.7 (38.5)	30.0 (18.9)	0.036
* Have you worried about the results of examination and tests?*	9 (90)	6 (60)	0.121
* Have you worried about your health in the future?*	9 (90)	9 (90)	1.0
M11: Shoulder problems, mean (SD) score	41.7 (41.8)	8.3 (16.2)	0.037
* Have you had problems raising your arm or moving it sideways?*	5 (50)	1 (10)	0.051
* Have you had pain in your shoulder?*	6 (60)	2 (20)	0.068
M12: Skin problems, mean (SD) score	16.7 (23.0)	97.8 (10.5)	0.287
* Have you had skin problems (e.g. itching, dry)?*	4 (40)	3 (30)	0.639
* Have you had a rash?*	1 (10)	0	0.305
* Has your skin changed colour?*	3 (30)	3 (30)	1.0
Single-item symptom			
S1: Coughing, mean (SD) score	16.7 (23.6)	40.0 (41.0)	0.140
* Have you had problems with coughing?*	4 (40)	6 (60)	0.371
S2: Opening mouth, mean (SD) score	43.3 (49.8)	36.7 (45.7)	0.759
* Have you had problems opening your mouth wide?*	5 (50)	5 (50)	1.0
S3: Social contact, mean (SD) score	7.4 (22.2)	0	0.347
* Have you had problems going out in public?*	1 (10)	0	0.279
S4: Neurological problems, mean (SD) score	50.0 (42.3)	13.3 (28.1)	0.035
* Have you had tingling or numbness in your hands or feet?*	7 (70)	2 (20)	0.025
S5: Swelling in the neck, mean (SD) score	26.7 (34.4)	3.3 (10.5)	0.066
* Have you had selling in your neck?*	5 (50)	1 (10)	0.051
S6: Weight loss, mean (SD) score	26.7 (37.8)	0	0.053
* Have you worried that your weight is too low?*	4 (40)	0	0.025
S7: Problems with wound healing, mean (SD) score	0	10.0 (22.5)	0.183
* Have you had problems with wound healing?*	0	2 (20)	0.136

Data as number of patients reporting all response options except for “not at all” and percentages in parenthesis unless otherwise stated

**Fig. 1:**
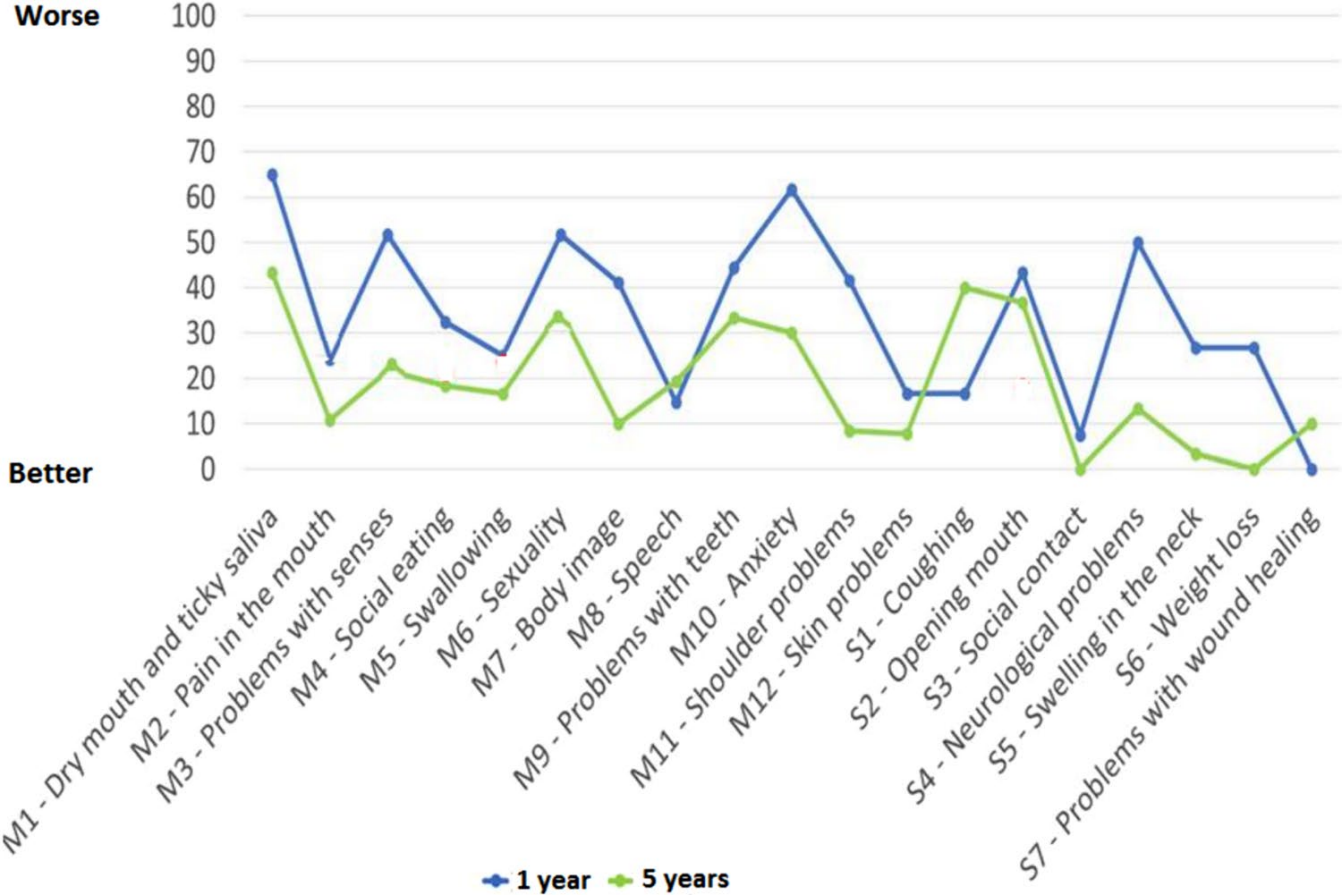
Mean values of the 12 multi-item scales of the 7 single-item symptoms of the EORTC QLQ-H and N43 questionnaire after 1 and 5 years of completing treatment for head and neck cancer

As shown in Fig. 1, almost all domains of the multi-item scales and single-item symptoms indicated better HRQoL after 5 years of treatment than after 1 year.

Finally, the EORTC QLQ-H&N43 questionnaire included five multi-item dimensions (dry mouth/sticky saliva, pain in the mouth, social eating, swallowing, and problems with teeth) and two single-item symptoms (opening mouth and problems with wound healing) focused on oral health, which would likely to be of interest to a dental provider.

### Feasibility of telephone interviews

The administration of the two FACT H&N and EORTC QLQ-H&N43 instruments by a professional interviewer was simple, easy, and feasible. None of the participants was excluded because of difficulties in completing the study questionnaires.

## Discussion

Head and neck cancer is a debilitating condition that not only affects physical health but also significantly impacts HRQoL of affected patients. Among the various challenges faced by those patients, dental care stands out as a crucial aspect due to the potential complications arising from cancer treatment, such as radiation therapy and chemotherapy [9]. This discussion explores the importance of dental care in enhancing the QoL of Head and Neck patients, highlighting the role of preventive measures, supportive care, and rehabilitation interventions.

The treatment modalities including surgery, radiation therapy, and chemotherapy, often result in adverse effects on oral health, such as xerostomia, mucositis, dental caries, and osteoradionecrosis. These complications not only cause physical discomfort but also affect essential functions like eating, speaking, and swallowing, thereby diminishing the overall HRQoL of patients [10, 11]. Moreover, the psychological impact of dental problems, such as self-consciousness about oral appearance and fear of social stigma, further exacerbates the burden on patients’ well-being.

Over the last decade, HRQoL has progressively become a more relevant outcome in oncological patients along with overall survival, disease-free survival, and toxicity after a multidisciplinary approach evaluation and combination of treatment modalities. The survival of head and neck is amplifying the importance of QoL. Problems with the mouth and teeth as side effects of treatment may disturb several important functions such as chewing, dry mouth, sticky saliva, and swallowing. These side effects may lead to inadequate nutrition as well as suboptimal aesthetic appearance and, thus, cause isolation and reduced HRQoL [12, 13]. Symptomatic relief measures, such as topical analgesics, saliva substitutes, and oral moisturizers, alleviate discomfort associated with xerostomia and mucositis, enabling patients to maintain oral function and nutritional intake. In fact, after head and neck cancer treatment, in particular cancer of the oral cavity, patients should receive routine dental care for prevention, treatment or minimizing complications affecting dental health [14, 15]. Orthodontics and restorative dentistry procedures may be essential components of the dental care needs of these patients [16].

Osteoradionecrosis may be a complication of tooth extraction in irradiated bone, usually the mandible [17]. Therefore, before starting radiotherapy it is advisable to check if any teeth require extraction, such as those partially erupted, with retained root tips, or periodontal involvement. It is important to establish an effective communication between the team members (medical and radio-oncologists, surgeons, odontologists) for appropriate assessment and decision-making. Before starting any oncological treatment, it is recommended to perform a complete oral and dental examination.

Most patients suffer from oral mucositis as an acute toxicity of cancer therapy, so that dental treatment cannot be carried out during the phase of active radiotherapy and chemotherapy as well as immediately after finishing these therapeutic modalities [18]. To reduce trauma and soft tissue irritation, irregular and rough dental surfaces should be eliminated [19]. Loss of taste is another common symptom in patients treated with radiotherapy. A study of the long-term outcome of radiotherapy-induced taste dysfunction demonstrated a decrease in the taste perception on the sweet, bitter, salty, acid and umami tastes during the first 6 weeks of treatment, with recovery of symptoms at 1 year from the beginning of radiotherapy [20].

Rehabilitation strategies aimed at restoring oral function and aesthetics are integral components of dental care for head and neck cancer survivors. Prosthodontic interventions, removable prostheses, and dental rehabilitation with maxillofacial prostheses, contribute to the restoration of oral function, speech intelligibility, and facial aesthetics, thereby enhancing patients’ psychosocial well-being and QoL. Speech therapy and swallowing rehabilitation programs address functional impairments resulting from HNC treatment, enabling individuals to regain confidence and independence in daily activities. Furthermore, multidisciplinary collaboration between oncologists, dentists, and allied healthcare professionals facilitate comprehensive care delivery, addressing the diverse needs of patients and optimizing their HRQoL outcomes [16].

Results obtained in the two main instruments specifically designed for measurement of HRQoL in head and neck cancer patients, the FACT H&N Symptom Index and the EORTC QLQ-H&N43 questionnaires, were in the same direction showing the impact of tumors and treatment with radiotherapy on patients’ HRQoL, being of a lower magnitude in patients evaluated at 5 years after treatment compared to those evaluated at 1 year. However, the appropriateness for dental treatment differs substantially between both instruments. While the FACT H&N Symptom Index only contains three items focused on oral health (swallow, pain in mouth, throat or neck, and solid foods), the EORTC QLQ-H&N43 has several multi-item scales measuring needs which could be alleviated through dental and orthodontics therapy, such as pain in mouth, social eating, swallowing, and particularly, problems with teeth.

The administration of the study questionnaires by an experienced telephone interviewer was simple and feasible. Equivalent results between telephone and patient-completed administration of HRQoL questionnaires including the EORTC QLQ-C30 for cancer patients, have been reported [21, 22].

Results of this study may be interpreted taking into account some limitations of the study especially the pilot characteristics based on a small sample size. Although the study questionnaires were administered when oncological treatment had been completed either at 1 or 5 years, other factors potentially related and unrelated to head and neck cancer that may affect HRQoL were not evaluated. The EORTC QLQ- H&N43 questionnaire was used in our study because it is a revised and updated version of the EORTC QLQ-H&N35 questionnaire, reflecting a more comprehensive assessment of the HRQoL of head and neck cancer patients with a broader range of symptoms and issues identified as relevant for these patients. Two of the eight new items incorporated in the EORTC QLQ- H&N43 refer directly to oral health, such as problems chewing and problems because of losing some teeth.

These preliminary results in patients with head and neck cancer after 1 and 5 years of completing oncological treatment showed that the impact on HRQoL measured using two specific instruments, the FACT H&N Symptom Index and EORTC QLQ- H&N43 questionnaires, is reduced when the follow-up is longer. The telephone administration of these questionnaires by a trained interviewer was simple and feasible. The EORTC QLQ-H&N43 questionnaire seemed to be more useful than the FACT H&N Symptom Index for assessing oral health.

## Conclusions

In conclusion, dental care plays a crucial role in enhancing the HRQoL of patients treated for head and neck cancer by addressing the oral complications associated with cancer therapy and facilitating rehabilitation interventions to restore oral function and aesthetics. By implementing preventive measures, supportive care interventions, and rehabilitation strategies, healthcare professionals can mitigate the adverse effects of cancer treatment on oral health and improve the overall well-being of head and neck cancer survivors.

## Data Availability

Study data are available from the corresponding author upon request.
